# Implementing Green Walls in Schools

**DOI:** 10.3389/fpsyg.2018.00619

**Published:** 2018-06-06

**Authors:** Michael B. McCullough, Michael D. Martin, Mollika A. Sajady

**Affiliations:** ^1^Project Sustainability, Environmental Education, Saint Paul, MN, United States; ^2^Department of Landscape Architecture, Iowa State University, Ames, IA, United States; ^3^Department of Developmental-Behavioral Pediatrics, University of Minnesota, Minneapolis, MN, United States

**Keywords:** nature-based learning, green walls, project-based learning, nature in the classroom, environmental education, living walls art and STEM

## Abstract

Numerous studies in applied pedagogical design have shown that, at all educational levels, direct exposure to the natural environment can enhance learning by improving student attention and behaviors. Implementing green walls—a “vertical garden,” or “living wall” interior wall that typically includes greenery, a growing medium (soil or substrate) and a water delivery system—provides environmental health benefits, but also provides a practical application within classrooms for minimizing directed attention fatigue in students by connecting them to “outdoor nature” within the indoor environment. Hands-on “project-based” learning is another pedagogical strategy that has proved to be effective across the spectrum of educational levels and across subject areas. Green walls have the potential to inspire critical thinking through a combination of project-based learning strategies and environmental education. The authors have outlined a curriculum involving the implementation of an indoor living wall system within a classroom-learning environment, incorporating project-based learning modules that interact with the wall. In conjunction with the passive health benefits of a green wall, project-based curriculum models can connect students interactively with indoor nature and have the potential to inspire real-world thinking related to science, technology, engineering, art, and mathematics fields within the indoor learning environment. Through a combination of these passive and interactive modes, students are connected to nature in the indoor environment regardless of weather conditions outdoors. Future research direction could include post-construction studies of the effectiveness of project-based curricula related to living walls, and the long-term impacts of implementing green walls in classrooms on school achievement and student behaviors.

## Introduction

In the United States, students spend an average of 6 to 7 hours per day in the classroom setting for approximately 180 days out of the year (Institute of Education Sciences, 2008). In an effort to improve standardized test scores, four out of ten schools nationwide have increased classroom instruction time in place of recess (Loucaides et al., 2009). Young learners are expected to maintain attention for prolonged hours during the school day and are more likely to suffer from directed attention fatigue, or decreased ability to stay focused on classroom activities. Attention restoration theory suggests that exposure to natural environments encourages involuntary attention, and these short breaks allow directed attention for the learning environment to reset (Kaplan, 1995).

A green wall, also known as a vertical garden or living wall, is defined as a vertical planting system that includes an integrated substrate, live plants, and in some cases an automated watering system. Implementing green walls in the classroom provides students with the opportunity to participate in passive, indirect attention, in order to have a restorative influence on focus and learning. There is limited evidence regarding the effect of incorporating nature into the classroom on student performance and behavior. One study of 170 Dutch students in grades five to seven found that children in classrooms with an indoor green wall had improved scores on a selective attention task and also rated their classroom more positively compared to children in classrooms without a green wall (Van den Berg et al., 2016). Another study in Taiwan found that junior high students in classrooms with plants had fewer sick days and misbehaviors, as well as higher reported measures of comfort and friendliness, compared to students in classrooms without plants (Han, 2009).

Stress recovery theory suggests that individuals have adapted over time to innately respond positively to natural environments, as opposed to built environments (Ulrich, 1983). In addition, plants have the potential to promote relaxation and recovery from stressful experiences. Visual access to nature from the classroom setting has been found to decrease attention deficit behaviors in children (Taylor et al., 2002). Views of nature from classroom windows have also been shown to improve directed attention skills compared to views of less natural scenes or of built environments (Tennessen and Cimprich, 1995). One randomized study of 94 public high school students in Illinois found that students assigned to classrooms with a “green” view scored higher on attention tasks and recovered from a stressful experience faster than students with no window or a window with a barren view (Li and Sullivan, 2016).

Academic achievement is positively influenced by exposure to nature in a variety of settings (McCormick, 2017). Vegetation surrounding schools is associated with improved standardized test scores on math and reading (Wu et al., 2014; Hodson and Sander, 2017). In addition, vegetation surrounding housing units in urban settings is associated with higher levels of cognitive functioning and attention scores among children (Wells, 2000; Taylor et al., 2002). Another study of 101 public high schools in Michigan found that views of greenery from classroom and cafeteria windows were associated with increased standardized test scores and graduation rates, in addition to decreased criminal behavior (Matsuoka, 2010).

Project-based, environmental education programs have been shown to increase student engagement, as well as improve academic performance in reading, writing, math, science, and social studies (Lieberman and Hoody, 1998; Chawla et al., 2014). To our knowledge, there is limited evidence regarding the implementation of green walls in schools as effective project-based learning curricula. The purpose of this article is to provide a practical application for implementing a nature-based learning curriculum into the classroom setting by utilizing green walls. By implementing green walls in classrooms, students can experience interactive learning through plant design, fabrication, and installation, and also gain passive exposure to nature. This can encourage environmental perspectives and minimize directed attention fatigue (Supplementary Figure 1).

## Using Nature and Project-Based Learning as a Strategy to Reduce Effects of Directed Attention Fatigue

Through a series of pilot programs that utilize multiple project-based learning strategies in combination with a green wall program, we have developed a model curriculum that provides students with passive indoor exposure to nature. The curriculum also features an interactive learning workshop that engages students with nature and with science, technology, engineering, art, and math (STEAM) fields. The outlined curriculum for the green wall workshop utilizes the concept of a simple plant-growing lab to create an environmental education collaboration. A living wall can be built on or within a preexisting building wall, or can be freestanding; bringing nature indoors in this way creates an intimate, ongoing exposure to living plants regardless of outdoor conditions and does not significantly reduce usable floor space.

The curriculum teaches the students about “vertical” gardens, and teaches them how to design and install the green wall. Passive exposure to the living wall is defined as presenting students with visual access to the green wall throughout the day. If a green wall is placed within an atrium or a library, students may have intermittent passive exposure; incorporating green walls in classrooms (where students spend a majority of their time) will allow for longer durations of exposure and afford a greater degree of interactivity with the wall. Green walls can conceivably be placed anywhere within the learning spaces inside a school.

## Green Wall Maker Workshop

The Green Wall Maker Workshop is a comprehensive approach for incorporating a green wall into a classroom, a project that allows students to participate in an interactive “design-build” experience that includes installation. As a broad-based project that incorporates both art and science, this workshop could be incorporated as a module within existing classes. The workshop is an example of “project-based learning,” a pedagogical strategy in the “active learning” mode that has its roots in the work of psychologist and educational reformer John Dewey (McDermott, 1981).

Active learning contrasts with passive modes of learning such as listening to lectures or reading assigned texts. In the passive mode, students receive information; in an active mode, students must seek information. Project-based learning is a form of active learning that causes the student to “learn by doing” and to apply ideas in purposeful fashion in order to complete a directed project. For project-based learning within a science course, for example, students might “investigate questions, propose hypotheses and explanations, discuss their ideas, challenge the ideas of others, and try out new ideas” (Krajcik and Blumenfeld, 2006). However, educators in any discipline can incorporate this strategy as a pedagogical device; it has been an essential component of design education for decades (Erdman et al., 2002). The essence of project-based learning is that the student *constructs* meaning and knowledge based on direct experience, interacting with their environment.

### Part 1: Study of Living Walls, Human Connection to Nature, and Plants

Part one of the workshop provides students with an opportunity to learn about how vegetation is brought indoors through methods related to STEAM. Students are given a 15-min presentation on how plants are used in architecture, and how creative process is the navigating tool for developing a green wall. This introductory presentation leads to hands-on learning through experimentation and one-on-one conversations with the green wall specialist (or other individual with an aptitude for the integration of plants within buildings). The objective of the specialist is to introduce plants as a component of interior environments, but also to inspire the students’ interest in education or career possibilities within the STEAM disciplines that underlie the concept of green walls.

Once the presentation is completed, students are introduced to physical attributes of interior plants. They study plant characteristics including form, color, texture, and scale. Students interpret these characteristics by designing a small-scale multimedia collage utilizing construction paper (in colors similar to the plants), drawing utensils (to simulate foliage textures), and adhesive. The objective is for students to understand that each plant have different characteristics, and those elements are used to create a planting design for the living wall (Supplementary Figure 2). During this process, students are encouraged to discuss the following questions, in order to maintain a focus on the purposefulness of their exercise: *Why bring plants indoors? How is there a disconnection between inside a classroom and the outdoors? How do you feel when you are playing in a park? Do you feel that way while in the classroom? How do plants provide oxygen?* Following the specialist’s presentation, studies of plants, and facilitated discussions, students will better understand how STEAM disciplines are used to bring plants into the built environment.

### Part II: Development of a Planting Plan

After students complete their collages, they then break into groups and develop a planting plan. If there is more than one green wall to create, groups may be split based on the number of installations. If there is only one green wall, the students participate in a design competition. All learners are encouraged to consider the characteristics of plants, to employ pertinent mathematical/geometrical concepts for pattern-making, and to collaborate in a creative process throughout all stages of the project.

The initial step for teams is to establish a design concept or theme that they intend to abstract in the pattern of the planting plan (the theme could be, for example, a process such as the flow of water, or a form such as the shape of a leaf). After they have arrived at a concept, the next step is to reconcile that theme within the limitations of a grid pattern, utilizing colored sheets of paper on a white board grid that is a reduced-scale version of the entire green wall (Supplementary Figure 3). This scale-model “mock-up” of the wall is readily manipulable, allowing team members to rearrange the pattern until the team achieves a consensus pattern that successfully abstracts the theme. This exercise employs three essential design-engineering processes: the translation of abstract concept to physical pattern, collaborative decision-making, and iterative design, during which the students consider alternative solutions.

Once Part II is completed, students will have engaged in conceptual thinking, developed a basic understanding of plant biology and geometrical pattern-making, and worked in collaboration to create a unique planting plan. Students will further understand how this same design process can be utilized to accomplish projects in other real-world scenarios.

### Part III: Fabrication of Living Walls and Plant Installation

Part three of the workshop, students may begin the formal fabrication the green wall(s) and subsequent plant installation. Some participating schools may not have the resources or requisite student skill sets to allow for the fabrication process to occur in the classroom. Alternatively, a carpenter, a skilled volunteer, or a living wall specialist could prefabricate the green wall(s), and the students begin their work with the installation of the plants that are represented in their respective planting plans. For schools that participate in the fabrication process, students engage in all construction stages and processes appropriate to their abilities, with guidance by the green wall specialist. Students may be engaged with all construction stages, from fastening the frame to the backing board to installing the unique planters specific to the respective workshop in preparation of plant installation (Supplementary Figure 4).

For the fabrication process, students are typically separated into teams that undertake particular tasks. During each step, students may discover construction challenges, such as ensuring the frame is square, securing and fastening the frame members, aligning the planters correctly, and physically attaching the green wall planters to the frame properly. These potential challenges often inspire discussion for collaborative problem-solving.

Once the frames of the green wall units are complete, the installation of plants begins, with the consensus pattern developed during the Part II exercise serving as the design template. The installation follows a pattern, but also includes crucial construction “detailing” that ensures the viability of the living system. Students must take care that the plants are installed with sufficient soil and that the plants are stabilized within the soil medium (Supplementary Figure 5). During the process, students are encouraged to occasionally break and review their progress to determine whether the green wall is being planted in accordance with their conceptual planting plan, or whether any modifications to the pattern might be desirable as the true-scale design comes into being. Once the students complete the plant installation, they assist with the clean up and preparation for the green wall(s) to be installed in their respective location(s).

Once Part III is completed, students will have learned about living wall fabrication processes, and will understand the relationship between the two-dimensional design plan (Part II) and the finished installation.

### Part IV: Living Wall Maintenance Training and Recurring Activities

Because green walls feature living plants, all installations require on-going plant care, watering, and maintenance of aesthetic qualities. To ensure the living walls thrive, students are encouraged to attend to each wall on a weekly basis as part of their art or science class, along with oversight by a committed community volunteer or green wall specialist. Students may apply critical thinking skills to individualize the care of each plant and identify when a plant is stressed. Through instructional oversight, students are provided with an introduction to plant care. Learners have hands-on experience to determine when a plant requires pruning, has too little or too much water, is infested with a pest that needs to be eradicated, or if the plant needs to be replaced. These recurring maintenance activities affords students the opportunity to continue their collaborative interactions as well as take responsibility for something meaningful, beyond what is offered by traditional classroom work (Ruiz-Gallardo et al., 2013).

## Other Learning Opportunities with the Living Wall Program

Throughout the entirety of the program, students are provided with an understanding of nature’s role in STEAM fields through presentation and project-based learning opportunities. The students use concepts of science through methods of understanding plant biology, capillary action, and plant care. The engineered plant container for the green wall (created by computer-aided drafting technology and 3-D printing) highlights for students some of the key technologies that underlie the green wall system. Engineering principles are emphasized for the design process of the planters, as well as the project-based learning activities with which the students are engaged during the creative planning and fabrication of the living walls. Mathematical and geometrical concepts are critical for understanding the quantities of plants used during the design phase, the dimensional requirements of each panel, and the number of planters needed for each panel. Finally, beyond technical considerations, creative or artistic process itself is essential to the project; students are directly engaged in the conversion of abstract concepts to built, functioning artifacts.

## Ensuring a Successful Installation

The long-term viability of the installation often depends on a volunteer supervisor who will ensure that students consistently maintain the green wall. The recurring plant care accomplished by the students under the trained supervision of a community volunteer, teacher, or a parent provides students with continued exposure to indoor nature, even during seasons when plants on the exterior may be dormant. Additionally, schools may experiment with growing certain varieties of edible plants (if sufficient lighting is available), which would enlarge the green wall concept to include potential for “vertical” indoor agriculture.

Most green walls in non-school institutional or commercial settings rely on maintenance by an outsourced Plantscape company, or by in-house trained horticultural technicians, which constitutes a significant ongoing expense. For schools, the authors propose that maintenance by students, staff or volunteers can obviate the involvement of outside agents, and that schools can seek funding for any ongoing or intermittent expenses through minor grants, artist-in-residency fees, or even through crowd-sourced funding. These strategies were essential to the implementation of the three pilot school projects mentioned in this paper.

## Conclusion and Future Direction

Using STEAM inspired project-based learning in this curriculum, students are provided with interactive and passive exposure to nature. This classroom model is a potential solution to reduce the effects of directed attention fatigue and improve student behavior by bringing nature indoors; at the same time, it affords students the opportunity to enhance their understanding of green technologies, build cooperative social skills, and develop design-process abilities by translating abstract concepts into built form—all of which is accomplished through directed, active-mode, project-based-learning. This active approach to learning has been associated with improved academic achievement within STEAM fields (Freeman et al., 2014). The students’ initiation with these technologies and processes at the elementary school level could potentially lead to their involvement in more sophisticated applications of green technologies in subsequent education levels, or perhaps inspire their interest in educational specialization within STEAM programs beyond high school.

Ongoing research and development of green wall systems will provide additional opportunities for incorporation of these technologies within classrooms, as environmental enhancement and as opportunity for other project-based learning models. Future research is needed to better understand and measure the effectiveness project-based green wall programs on student learning and well-being, at various educational levels.

## Author Contributions

MBM was involved in curriculum design, education outreach, classroom pilot implementation, and manuscript writing. MS was involved in curriculum design and manuscript writing. MDM was involved with curriculum design and manuscript writing.

## Conflict of Interest Statement

The authors declare that the research was conducted in the absence of any commercial or financial relationships that could be construed as a potential conflict of interest.

## Abbreviations

STEAM: Science, Technology, Engineering, Art, and Mathematics
