# Supplementary material for: Implementing Green Walls in Schools
Source: Front Psychol. 2018 Jun 6;9:619. doi: 10.3389/fpsyg.2018.00619 (PMC5997894; doi:10.3389/fpsyg.2018.00619)

Supplementary Material

Implementing Green Walls in Schools

Michael B. McCullough*, Mollika A. Sajady, Michael D. Martin

*** Correspondence:** Michael B. McCullough, mbmccull@gmail.com

- 1. **Supplementary Figure 1.** Green wall in an interior atrium of elementary school in Edina, Minnesota provides passive exposure to nature as well as interactive learning for students. Written permission for the publication of this figure was obtained from students’ parents.

#
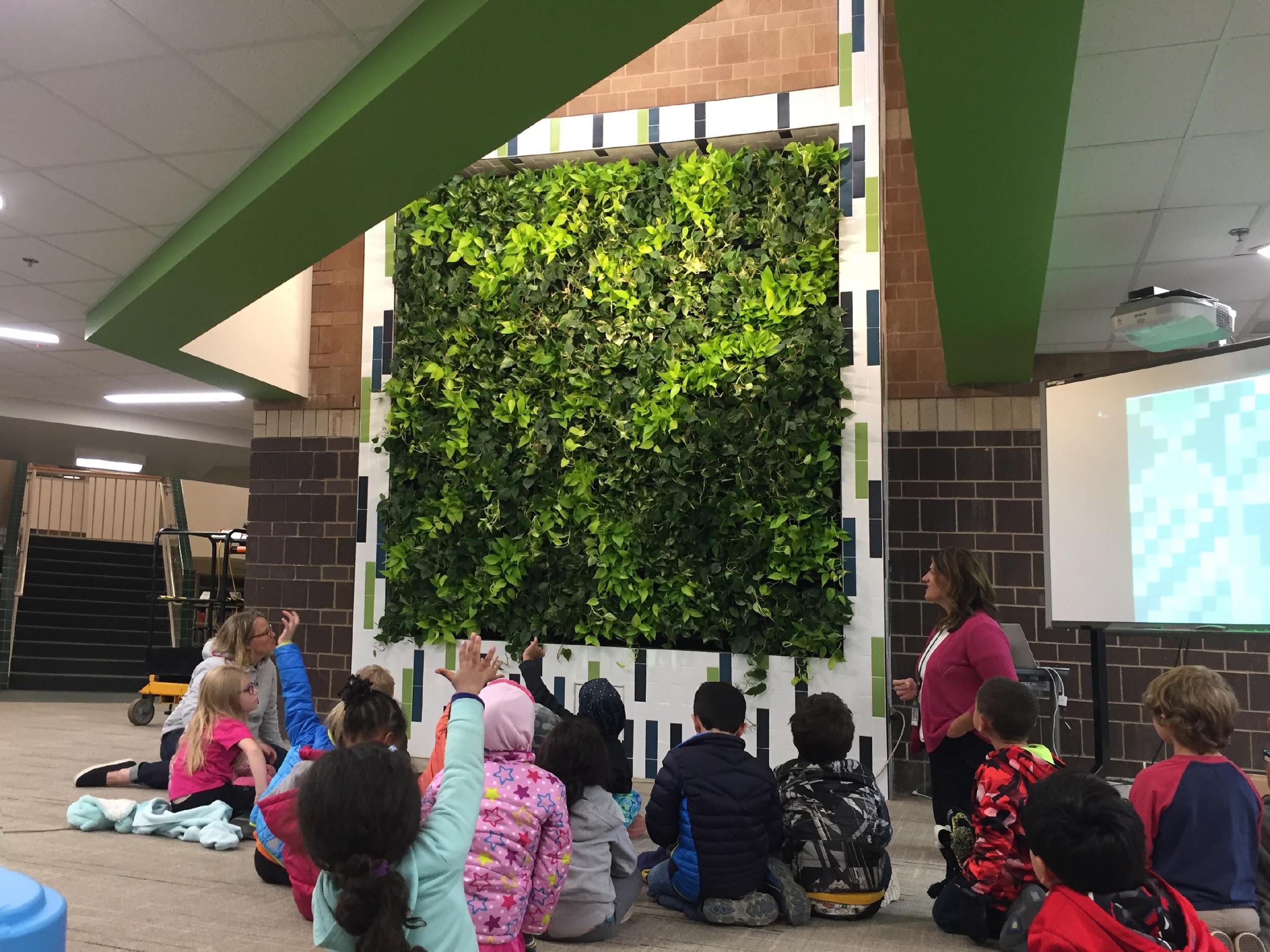


- 1.
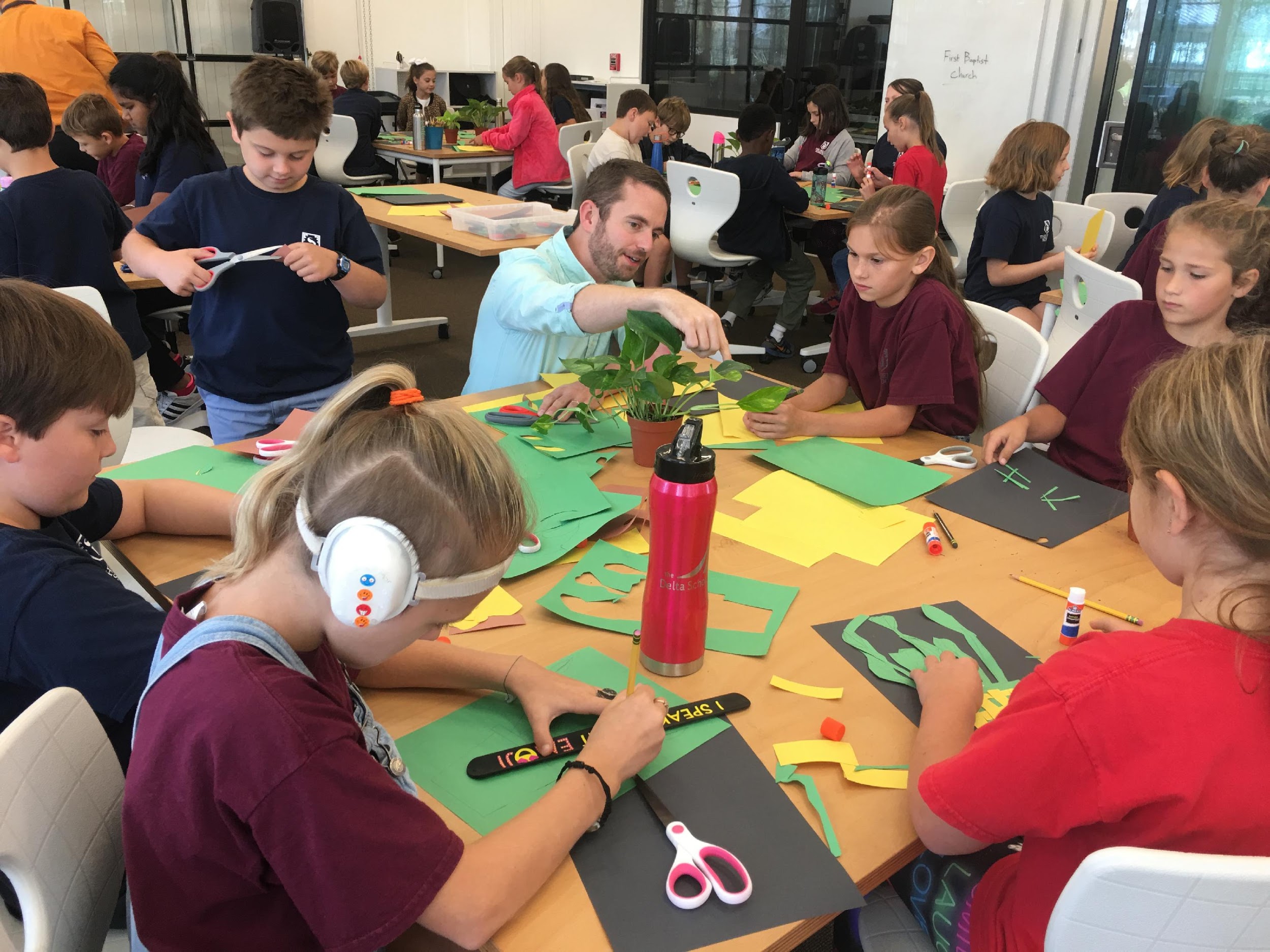
**Supplementary Figure 2.** Students study plants that are used in a green wall through the creation of a paper collage and drawing. Written permission for the publication of this figure was obtained from students’ parents.
  2. **Supplementary Figure 3.** Students collaborate on the creation of a planting plan by designing a colored paper grid that corresponds to the planters available in each green wall. Written permission for the publication of this figure was obtained from students’ parents.
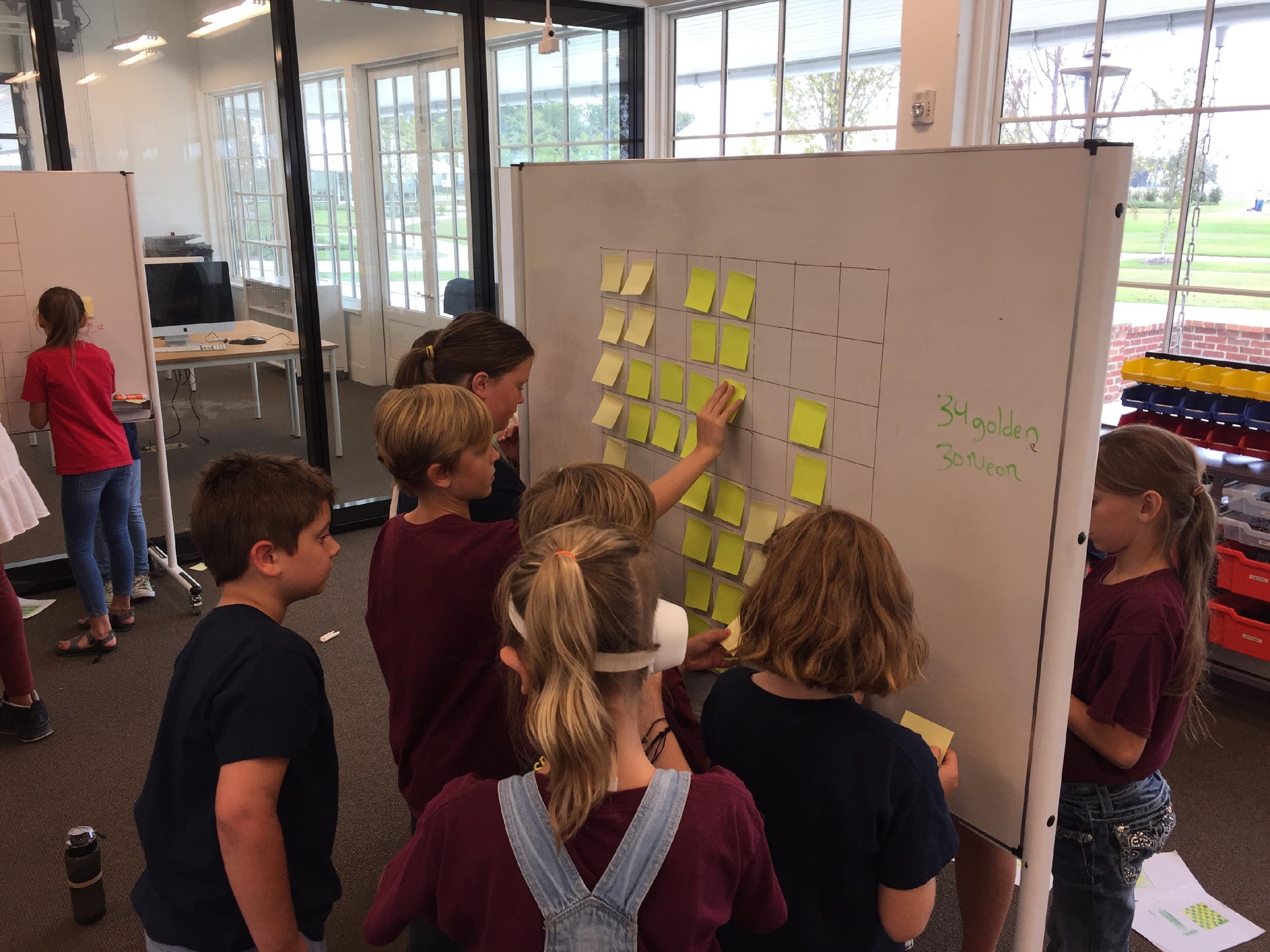

  3. **Supplementary Figure 4.** Students installing the planters onto the frame. Written permission for the publication of this figure was obtained from students’ parents.
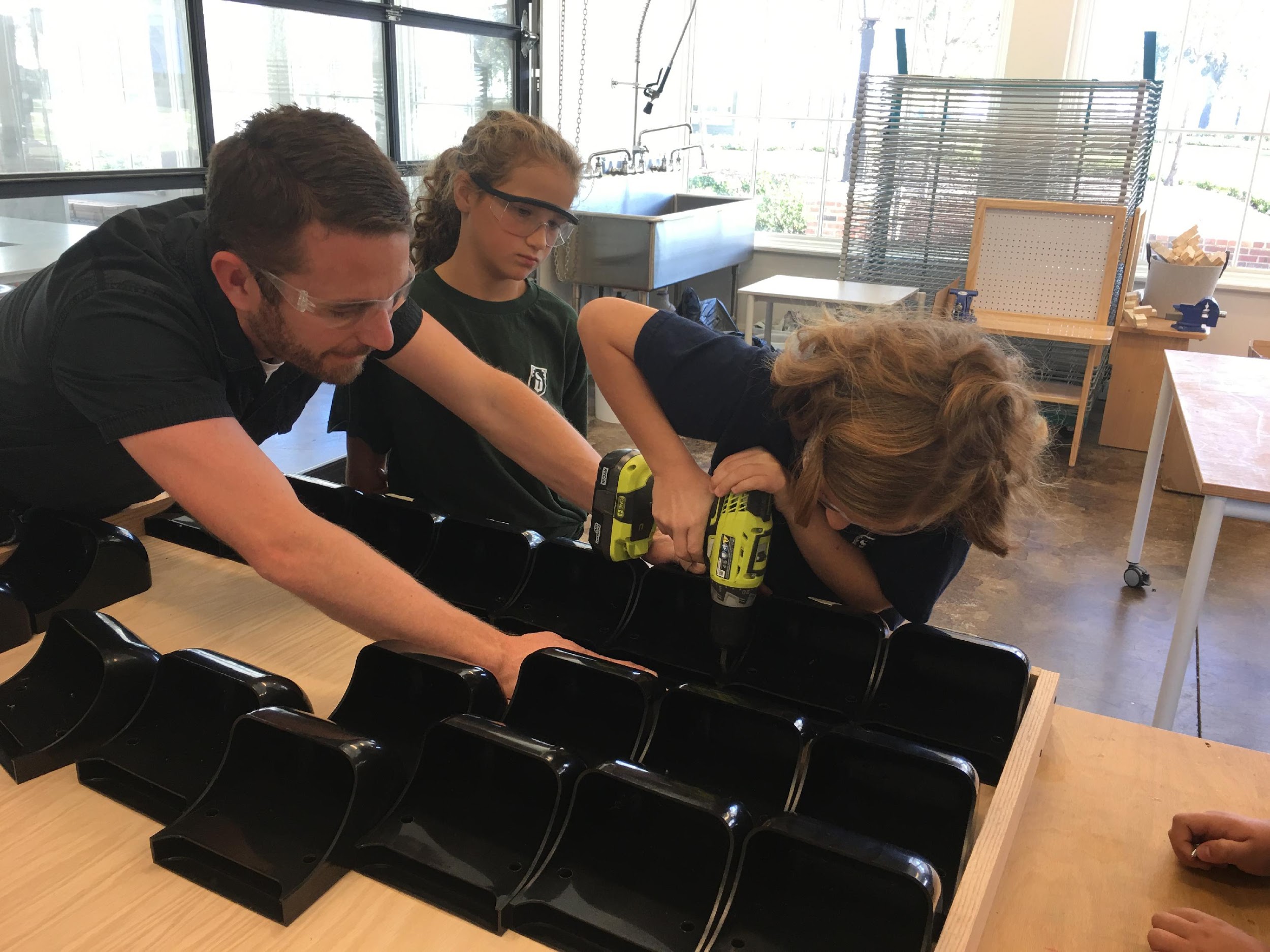

  4. **Supplementary Figure 5.** Students installing plants based on the planting plans they collaboratively developed in Part II of the Green Wall Maker Workshop. Written permission for the publication of this figure was obtained from students’ parents.


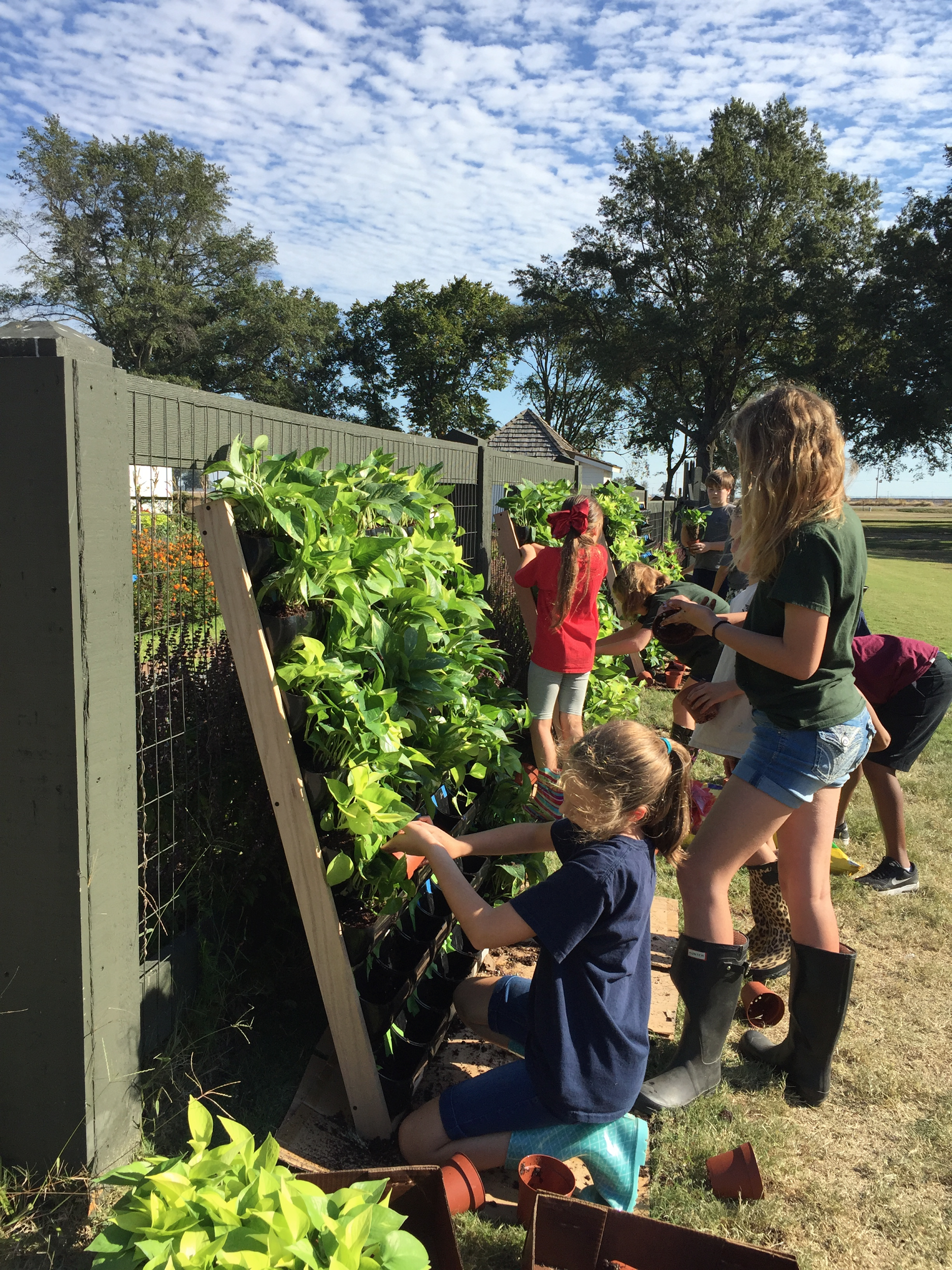

Supplement: Supplementary file 1 [file Data_Sheet_1.DOCX]
